# Assessment of AI-based cephalometric landmark recognition on lateral cephalometric radiographs

**DOI:** 10.3389/froh.2026.1889431

**Published:** 2026-08-07

**Authors:** Amani Idres, Mohamed Osman, Ahmed Ghoneima

**Affiliations:** 1 Department of Orthodontics and Pediatric Dentistry, Hamdan Bin Mohammed College of Dental Medicine, Dubai, United Arab Emirates; 2 Independent Researcher, Calgary, AB, Canada; 3 Section of Orthodontics, UCLA School of Dentistry, Los Angeles, CA, United States

**Keywords:** anatomic landmarks, artificial intelligence, cephalometric analysis, orthodontics, reliability

## Abstract

**Background:**

Artificial intelligence (AI) strategies have been proposed for automatic landmark recognition applications with the expectation of simplifying and improving cephalometric analysis through accurate and consistent landmarks identification. The aim of the current study was to evaluate the accuracy and reliability of AI in identifying cephalometric landmarks for orthodontics cephalometric tracing.

**Materials and methods:**

A total of 506 lateral cephalometric radiographs of patients aged 14–40 years were retrieved from the archives of Dubai Dental Hospital from the period of 2018–2021. Radiographs were traced using Dolphin imaging software, and the AI program (AI Ceph) was trained to identify 19 key cephalometric landmarks. Manual tracings were compared with AI-generated ones to evaluate the accuracy and reliability of the AI Ceph program.

**Results:**

High agreement was observed between manual and AI-generated tracings. The highest concordance was detected for Menton (96.6%), whereas the lowest was observed for Nasion (86.6%). The deviation between the two methods was within 2 mm for all landmarks except Glabella (*p* < 0.02). Sensitivity values exceeded 80% for all evaluated landmarks except for Nasion and A-point where they showed a sensitivity of 72.7% and 78.8% respectively, demonstrating acceptable accuracy and reliability of the AI system.

**Conclusions:**

The tested AI method was accurate and reliable for identifying cephalometric landmarks, with a clinically acceptable 2 mm deviation. AI-assisted cephalometric tracing may serve as a useful adjunctive tool to support orthodontists in diagnosis and treatment planning.

## 1 Introduction

Artificial Intelligence (AI) is described as the capability of machines to imitate human reasoning and carry out difficult tasks (1). The use of AI in lateral cephalometric analysis in orthodontic imaging is one of the remarkable developments (2–4). Within the dental field, AI algorithms can be useful in the localization of cephalometric landmarks, the identification of dental decay and periapical or periodontal lesions and defining maxillofacial cysts and tumours. For example, convolutional neural networks (CNNs), deep learning (DL) algorithms and support vector machines (SVMs) have been used to process large set of cephalograms and classify specific anatomical structures previously. Random forest classifiers are also used in cephalometric analysis, presenting a decision-tree-based method to predict and classify anatomical locations based on different input features (5, 6).

The process of orthodontics diagnosis and treatment planning requires detailed analysis of multiple diagnostic records including lateral cephalometry that helps review the relationship between the bones and teeth and monitor growth and treatment-related changes (7–9). Traditionally, anatomic landmark identification in lateral cephalometric imaging has been performed manually by trained orthodontists, which is considered the gold standard for accuracy and precision in cephalometric analysis. However, this manual process is slow and prone to variability within and between observers (10).

Previous studies have investigated automated identification of cephalometric landmarks on lateral cephalometric radiographs. The transition from conventional manual cephalometric tracing to AI-based analysis is expected to enhance diagnostic efficiency by reducing tracing errors and minimizing the time required for analysis (11–13). Arik et al. utilized CNNs for landmark identification on lateral cephalograms (14). Subsequently, Park and Hwang applied DL techniques using a dataset of 1,028 cephalograms. In addition, Kunz et al. and Yu et al. evaluated the application of AI in orthodontics, including the accuracy of AI-generated cephalometric measurements (12, 15, 16). The aim of the present study was to assess the accuracy and reliability of the specifically designed AI Ceph program in identifying and localizing anatomical landmarks on lateral cephalometric radiographs.

## 2 Materials and methods

### 2.1 Study design and ethical approval

This cross-sectional study was approved by the Institutional Review Board (IRB) of Mohammed Bin Rashid University of Medicine and Health Sciences. A total of 506 lateral cephalometric radiographs were retrieved from the archives of Dubai Dental Hospital. All radiographs were anonymized to ensure patient confidentiality, and the data were securely stored on an encrypted hard drive.

### 2.2 Sample selection

A power analysis was conducted prior to the study and indicated that a minimum sample size of 402 cephalometric radiographs was required to achieve a statistical power of 0.80. The selected radiographs were obtained between 2018 and 2021 from patients who underwent orthodontic treatment at the hospital clinics. The study cohort included individuals aged 14–40 years, comprising 251 females and 255 males. Radiographs were excluded if patients presented with cleft lip and palate, other craniofacial anomalies, previous orthognathic surgery, absence of first molars or central incisors, or obvious radiographic artifacts that could compromise landmark identification.

### 2.3 Image acquisition and landmark selection

**Figure 1:**
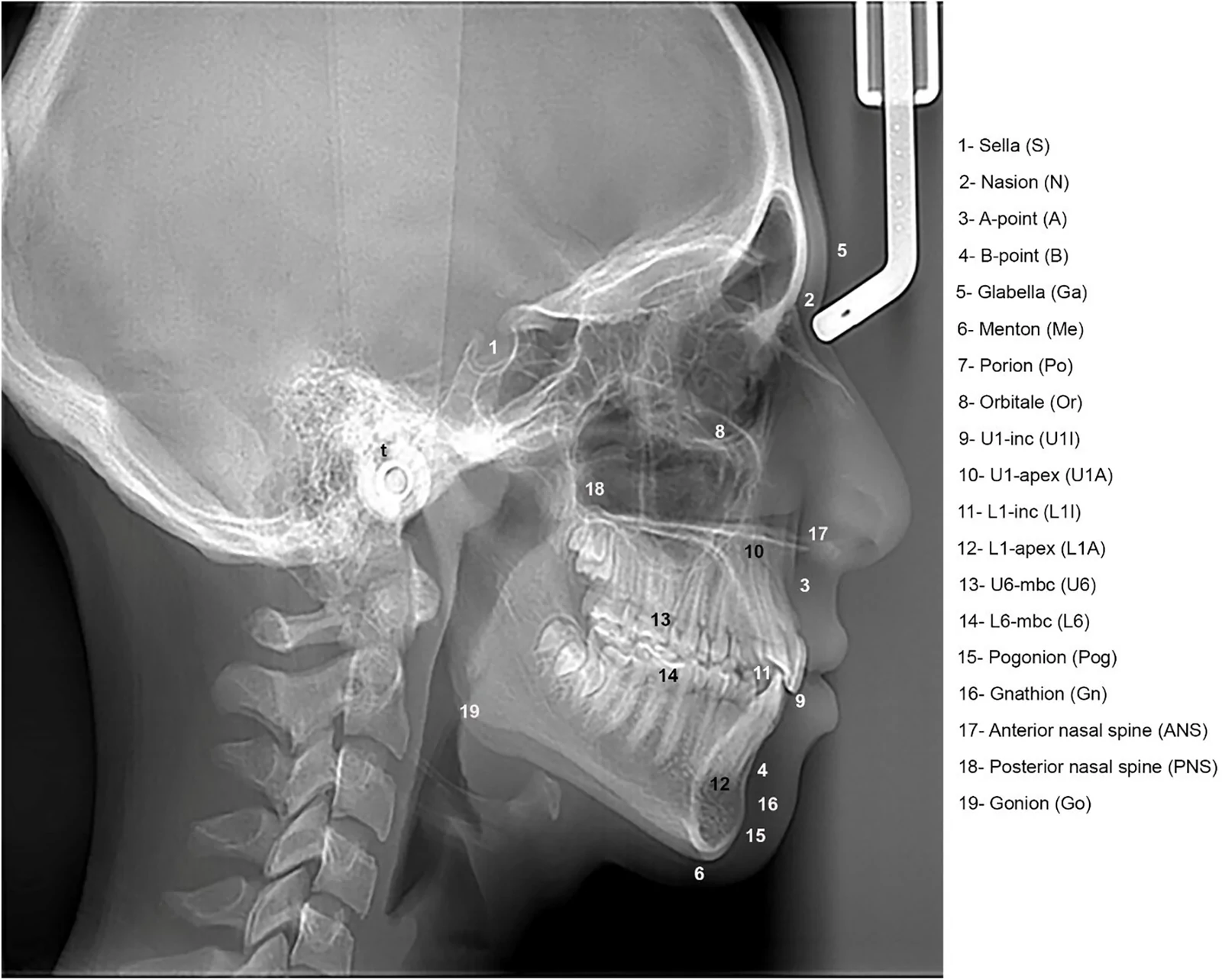
The cephalometric landmarks selected in the study.

All cephalometric radiographs were captured using a Soredex CRANEXr Excel Ceph digital x-ray machine (Tuusula, Finland) and saved in JPG format. Image processing was performed using Soredex SorCom software (version 3.1.5, version 2.0). The image resolution was 1,935 × 2,400 pixels with a pixel spacing of 0.1 mm. All radiographs were uploaded into Dolphin Imaging software (version 11.95; Dolphin Imaging, Chatsworth, CA, USA) for cephalometric tracing and landmark identification. Nineteen cephalometric anatomical landmarks were selected for analysis, including 18 hard tissue landmarks and one soft tissue landmark (Table 1, Figure 1). Radiographs were presented to observers in a randomized order to minimize observational bias.

**Table 1:** Definition of anatomical landmarks used in the study.

Landmark	Abbreviation	Definition
Sella	S	The center of the pituitary fossa of the sphenoid bone was determined by inspection.
Nasion	N	The junction of the frontonasal suture at the most posterior point on the curve at the bridge of the nose.
A-point	A	The most posterior point on the curve of the maxilla between the anterior nasal spine and supradentale.
B-point	B	The point most posterior to a line from infradentale to pogonion on the anterior surface of the symphyseal outline of the mandible; the B-point should lie within the apical third of the incisor roots.
Glabella	Ga	The height of curvature of the bone overlying the frontal sinus; in cases where this point is not readily apparent, the overlying soft tissue is used to locate it.
Menton	Me	The most inferior point on the symphyseal outline.
Porion	Po	The midpoint of the line connects the most superior point of the radiopacity generated by each of the two ear rods of the cephalostat. The point on the upper margin of the porous acusticus externus.
Orbitale	Or	The lowest point on the average of the right and left borders of the bony orbit
Upper central incisal edge	U1I	The incisal tip of the maxillary central incisor
The upper central root apex	U1A	The root tip of the maxillary central incisor
Lower central incisal edge	L1I	The incisal tip of the mandibular central incisor
The lower central root apex	L1A	The root tip of the mandibular central incisor
Upper first molar mesial cusp	U6	Maxillary first molar mesial cusp
Lower first molar mesial cusp	L6	Mandibular first molar mesial cusp
pogonion	Pog	Most anterior points on the symphysis of the mandible in the median plane when the head is viewed in Frankfort relation
Gnathion	Gn	The lowest, most anterior midline point on the symphysis of the mandible
Anterior nasal spine	ANS	The tip of the median, the sharp bony process of the maxilla at the lower margin of the anterior nasal opening
Posterior nasal spine	PNS	The most posterior point at the sagittal plane on the bony hard palate
Gonion	Go	The external angle of the mandible, located on the lateral radiograph by bisecting the angle formed by tangents to the posterior border of the ramus and the inferior border of the mandible (a line from menton to the poster inferior border of the mandible)

### 2.4 AI training and landmark detection

Each cephalometric radiograph was traced twice by the same investigator: once using the conventional tracing function available within Dolphin Imaging software and once using a deep learning (DL)-based artificial intelligence augmentation method (AI Ceph).

**Figure 2:**
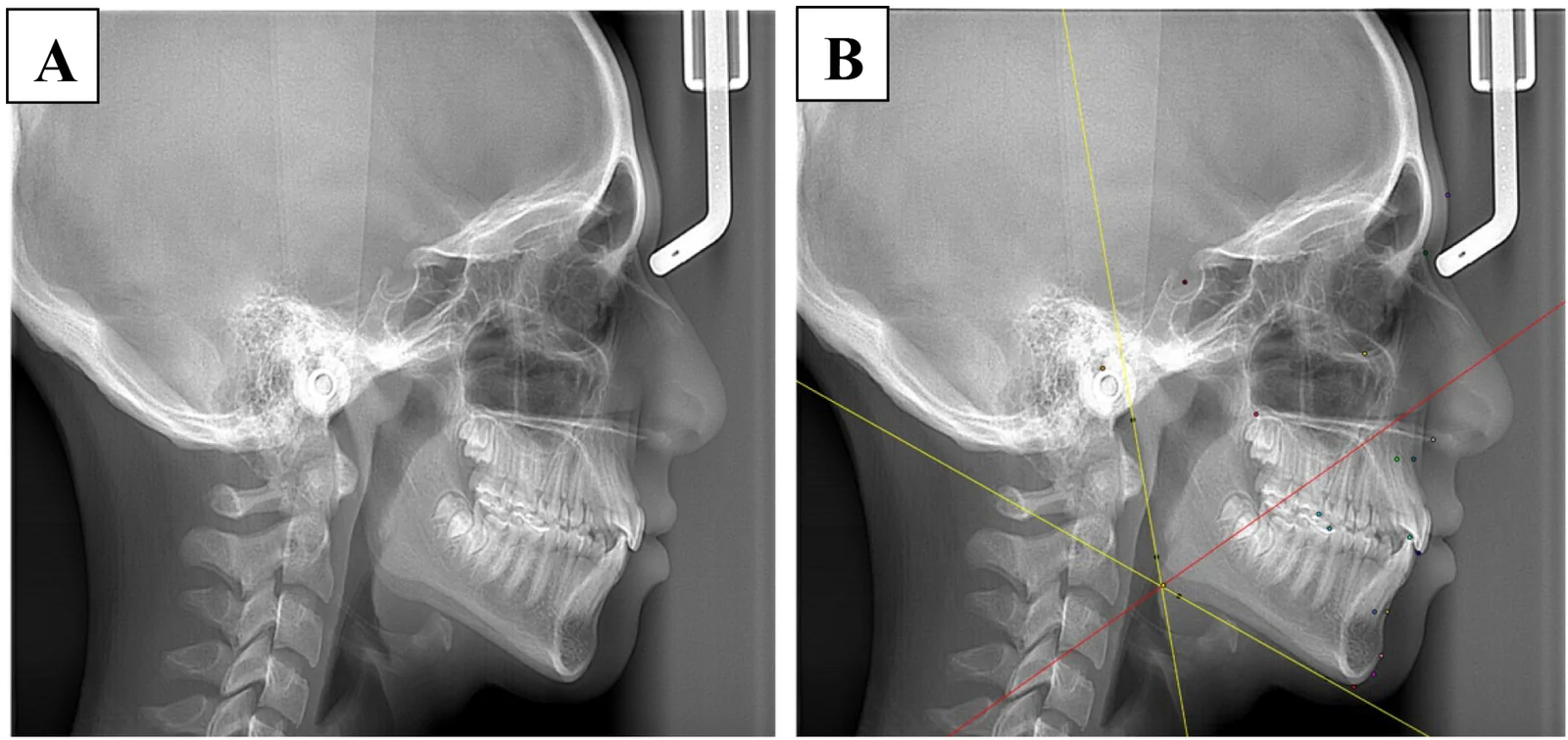
**(A)** Lateral cephalometric x-ray. **(B)** AI training to identify the points in the cephalometric training phase.

The AI Ceph model was developed in-house using a DL framework specifically designed for cephalometric landmark detection (Figure 2). Initially, the AI model automatically identified the landmarks, after which the observer adjusted the landmark positions when necessary. The DL AI/human augmentation tracing procedure was repeated after a 1-week interval in randomized order until the AI model was able to independently identify all landmarks without further manual adjustments. The AI model was developed using 440 cephalometric radiographs for model training, while a separate set of 33 radiographs was allocated for independent evaluation of the agreement between AI-generated and manual landmark identification.

### 2.5 Neural network architecture

The neural network was developed using a Feature Pyramid Network (FPN) architecture, which enables preservation of both fine-scale and large-scale image features. This architecture was selected because cephalometric landmark prediction requires integration of detailed local anatomical structures with broader craniofacial contextual information.

Unlike conventional convolutional or transformer-based image classification networks, which progressively compress image details into generalized representations, the FPN architecture maintains high-resolution spatial information essential for precise coordinate prediction tasks. Transfer learning was implemented using an ImageNet-pre-trained ResNet-50 backbone within the FPN architecture. The model was trained using an L2 loss function to minimize prediction errors.

Prior to training, all cephalometric radiographs were resized, converted to grayscale, normalized, and downsampled to a model input resolution of 224 × 224 pixels. Data augmentation was applied only to the training dataset and included random brightness and contrast adjustment, rotation, affine transformation, and grid distortion, with corresponding landmark coordinates transformed simultaneously to preserve spatial alignment. The model was trained for 100 epochs using the Adam optimizer (initial learning rate: 1 × 10^−5^; batch size: 2). The learning rate was automatically reduced when validation performance plateaued, and the final model was selected based on the lowest validation mean absolute error (MAE). Model development and training were implemented using the PyTorch and PyTorch Lightning frameworks.

### 2.6 Validation and concordance assessment

Following training and validation, 33 previously unseen cephalometric radiographs were used to evaluate concordance between AI-generated and manual tracings. In this phase, radiographs were initially traced by the AI model and subsequently traced manually by the investigator.

**Figure 3:**
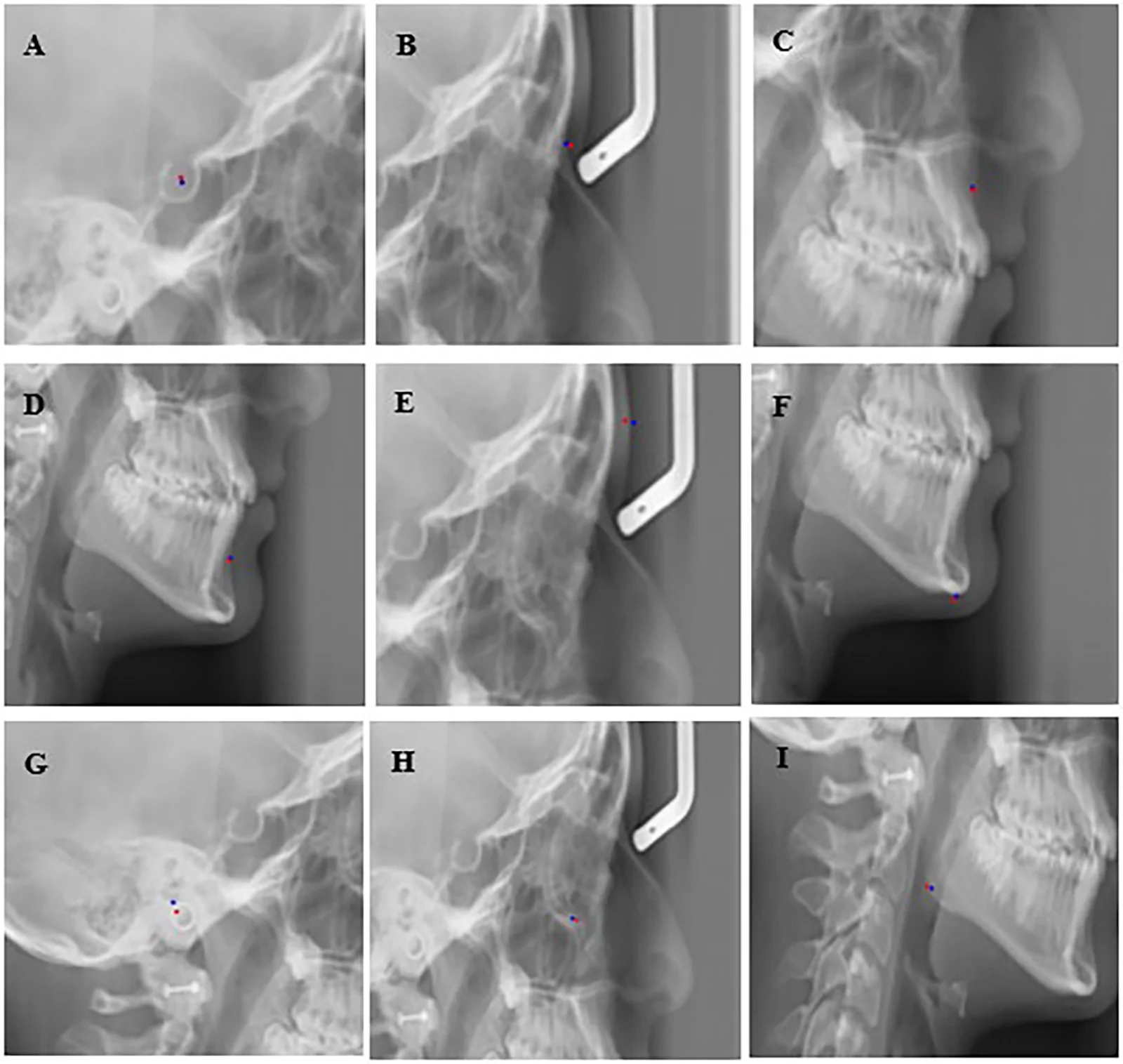
**(A)** Point Sella superimposition, a blue colored dot is manually selected, and a red dot is AI selected. **(B)** Point Nasion superimposition. **(C)** Point A superimposition. **(D)** point B superimposition. **(E)** Point Glabella superimposition. **(F)** Point menton superimposition. **(G)** Point Porion superimposition. **(H)** Point orbitale superimposition. **(I)** Point gonion superimposition.

**Figure 4:**
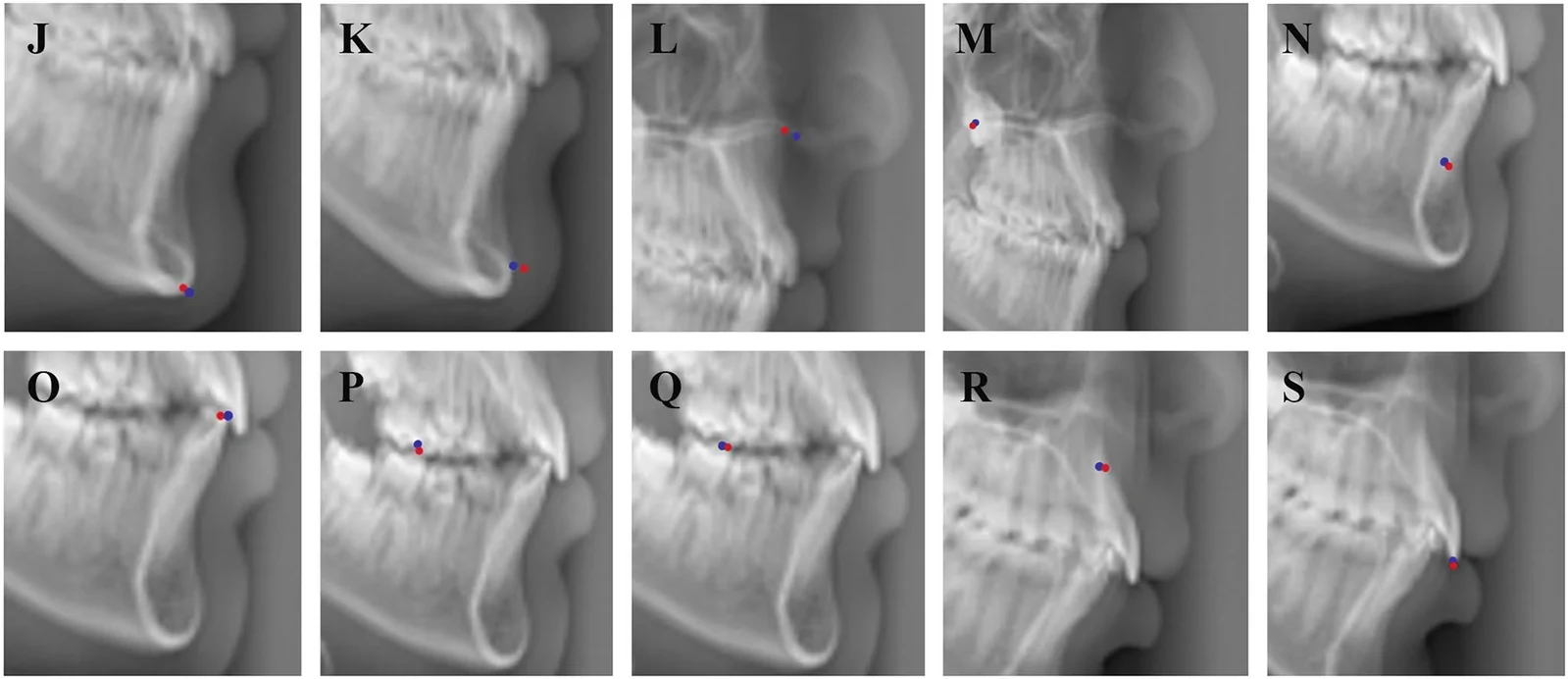
**(J)** Point Gnathion superimposition, a blue colored dot is manually selected, and a red dot is an AI selected. **(K)** Point pogonion superimposition. **(L)** Point ANS superimposition. **(M)** Point PNS superimposition. **(N)** Point lower central apex superimposition. **(O)** point Lower central incisal edge superimposition. **(P)** Upper first molar U6 superimposition. **(Q)** Lower first molar L6 superimposition. **(R)** Upper central apex superimposition. **(S)** Upper central incisal edge superimposition.

Manual and AI-generated tracings were superimposed using Microsoft PowerPoint 365 (Microsoft, Redmond, WA, USA). To preserve image integrity, the aspect ratio of both tracings was locked to prevent distortion. The transparency of the manual tracing was reduced by 50% before overlaying it onto the AI-generated tracing. Alignment was performed using a best-fit approach based on stable anatomical structures, including the cranial base, zygomatic bone, mandibular lower border, and soft tissue profile (Figures 3, 4). To assess the proximity between AI-generated and manually identified landmarks, a circular marker with a fixed diameter of 2 mm was superimposed at each landmark location. The manual reference standard used for comparison with the AI-generated landmarks was established by the primary investigator (A.I.). The second investigator (A.G.) independently identified the landmarks only for the assessment of inter-rater reliability and was not involved in generating the reference standard. Consequently, the reference landmarks were based on a single examiner and were not derived by consensus or averaging of coordinates.

### 2.7 Statistical analysis

For intra- and inter-rater reliability assessment, all cephalometric landmarks were identified twice with a 1-week interval by a trained investigator (A.I.) and independently repeated by an experienced investigator (A.G.). Data analysis was performed using IBM SPSS Statistics for Windows version 29.0 (IBM Corp., Chicago, IL, USA). Continuous variables were summarized using measures of central tendency and dispersion, whereas categorical variables were presented as proportions and percentages. The Kolmogorov–Smirnov test was used to assess the normality of continuous variables. Intra- and inter-rater reliability analyses were conducted using paired *t*-tests or Wilcoxon signed-rank tests depending on data distribution. Statistical significance was established at *p* < 0.05.

## 3 Results

### 3.1 Dataset characteristics and model development

Levene's test confirmed equal variances across the evaluated samples. The AI Ceph program identified 19 cephalometric landmarks from a dataset comprising 506 subjects. Coordinates corresponding to each landmark were recorded to establish a labelled dataset. Of the total radiographs, 440 images were allocated for model training, 33 for validation, and 33 for testing.

### 3.2 Concordance between manual and AI tracing

Overall, a high level of agreement was observed between the gold standard manual tracing method and the AI-generated tracings produced by the AI Ceph program.

The highest concordance rate was detected for Menton (96.6%), whereas the lowest concordance was observed for Nasion (86.6%). Three landmarks, Sella (89.3%), Nasion (86.6%), and Glabella (89.5%), demonstrated concordance rates below 90%, while all remaining landmarks showed concordance values exceeding 90%. The highest concordance rates were recorded for Menton (96.6%), L6 (96.5%), B-point (96.1%), and Gonion (95.3%) (Table 2). The maximum deviation between the corresponding landmark locations identified by the manual and AI tracing methods remained within a clinically acceptable range of 2 mm.

**Table 2:** Concordance pair between manual and AI tracings.

Landmark	% Concordance pair
Sella	89.3
Nasion	86.6
A point	93.1
B point	96.1
Glabella	89.5
Menton	96.6
Porion	93.7
Orbitale	94.7
U1i	90.3
U1a	93.5
L1i	93.3
L1a	91.7
U6	93.9
L6	95.5
Pogonion	94.3
gnathion	92.5
ANS	93.5
PNS	91.3
Gonion	95.3

### 3.3 Diagnostic performance of the AI model

To further assess the performance of the AI method relative to the manual tracing, sensitivity, specificity, positive predictive value (PPV), and negative predictive value (NPV) were calculated using 66 labelled records, including 33 correctly labelled and 33 incorrectly labelled cephalometric landmarks.

The highest sensitivity was observed for Gonion (100%), whereas sensitivity values exceeded 80% for most landmarks. The lowest sensitivity values were recorded for Nasion (72.7%) and A-point (78.8%). Specificity, which reflected the AI model's ability to correctly identify incorrectly labelled landmarks, ranged from 18.7% for A-point to 93.7% for posterior nasal spine (PNS) (Table 3).

**Table 3:** Performance of AI-based cephalometric landmark identification compared with manual tracing.

Landmark	Sensitivity	Specificity	PPV	NPV	% of Concordance
Sella	81.8	78.1	80.4	71.4	90.8
Nasion	72.7	62.5	70.7	58.4	86.2
A point	78.8	18.7	48.4	36.3	89.2
B point	90.9	68.7	82.3	76.1	95.4
Glabella	97.0	71.9	85.5	90.1	98.4
Menton	93.9	81.2	86.8	85.9	96.9
Porion	93.9	71.9	84.2	82.9	96.9
Orbitale	84.8	68.7	79.2	68.9	92.3
U1i	84.8	62.5	78.6	64.9	92.3
U1a	90.9	56.2	81.3	68.7	95.3
L1i	97.0	84.4	89.1	92.6	98.5
L1a	87.9	87.5	87.9	80.2	93.9
U6	87.9	87.5	87.9	80.2	93.9
L6	90.9	37.5	63.0	67.6	95.4
Pogonion	90.9	60.0	68.8	78.6	95.4
Gnathion	90.9	50.0	65.2	74.8	95.4
ANS	97.0	68.7	85.0	89.3	98.5
PNS	90.9	93.7	93.3	85.2	95.4
Gonion	100	90.6	93.0	100	100

The highest PPV values were observed for Gonion (93.0%) and Sella (80.4%), indicating strong predictive performance of the AI model when identifying positive landmarks correctly. In contrast, the lowest PPV was recorded for A-point (48.4%). Similarly, the highest NPV was observed for Gonion (100%), while lower NPV values were noted for Nasion (58.4%) and Sella (71.4%). Despite variability among certain landmarks, the overall concordance between AI-generated and manual tracings remained above 90% for most cephalometric landmarks (Table 3).

### 3.4 Effect of training with mislabelled landmarks

**Figure 5:**
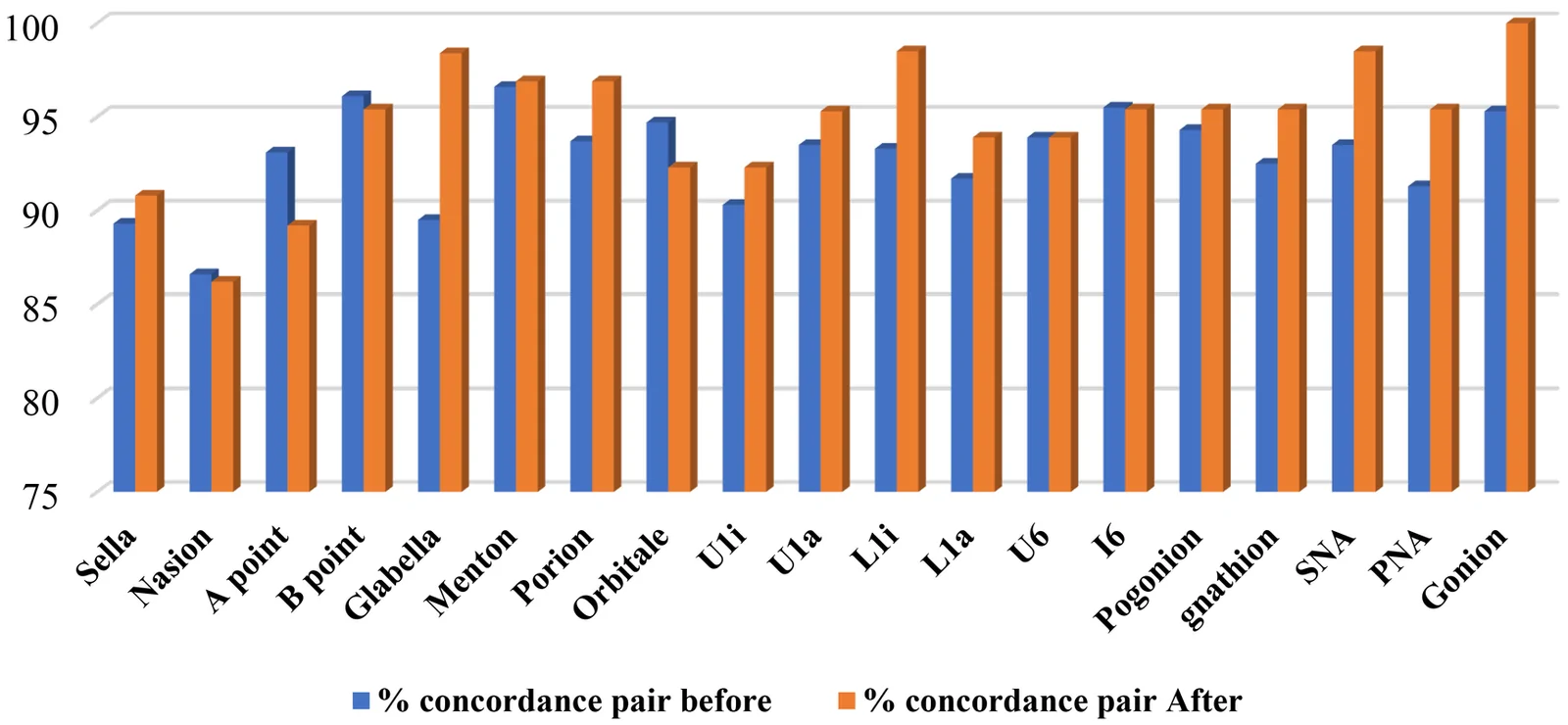
Concordance of the selected landmarks before and after training.

Training the AI model using mislabelled landmarks demonstrated no statistically significant changes in concordance rates for most evaluated landmarks. However, Glabella showed a statistically significant improvement in concordance following training (*p* = 0.02) (Figure 5).

Several landmarks, including Sella, Menton, Porion, U1i, U1a, L1i, L1a, Pogonion, Gnathion, anterior nasal spine (ANS), posterior nasal spine (PNS), and Gonion, demonstrated slight increases in concordance percentages after training with mislabelled landmarks; however, these improvements did not reach statistical significance (Table 4).

**Table 4:** Comparison between the percentage of the concordance pairs before and after training.

Landmark	% Concordance pair before training	% Concordance pair after training	Difference (after—before)	*p*-value
Sella	89.3	90.8	1.5	0.71
Nasion	86.6	86.2	−0.4	0.93
A point	93.1	89.2	−3.9	0.26
B point	96.1	95.4	−0.7	0.79
Glabella	89.5	98.4	8.9	0.02*
Menton	96.6	96.9	0.3	0.93
Porion	93.7	96.9	3.2	0.30
Orbitale	94.7	92.3	−2.4	0.43
U1i	90.3	92.3	2	0.60
U1a	93.5	95.3	1.8	0.57
L1i	93.3	98.5	5.2	0.10
L1a	91.7	93.9	2.2	0.54
U6	93.9	93.9	0	1.00
L6	95.5	95.4	−0.1	0.97
Pogonion	94.3	95.4	1.1	0.72
gnathion	92.5	95.4	2.9	0.39
ANS	93.5	98.5	5	0.11
PNS	91.3	95.4	4.1	0.26
Gonion	95.3	100	4.7	0.07

* Significant at *p* ≤ 0.05.

Overall, the findings indicate that the effect of training with mislabelled landmarks varied among different anatomical points. For most cephalometric landmarks, no statistically significant differences in agreement were observed before and after training. Glabella was the only landmark demonstrating a statistically significant improvement following additional AI training.

## 4 Discussion

AI has increasingly contributed to the field of orthodontics through a wide range of applications, including assessment of facial attractiveness from photographs, analysis of skeletal relationships, and prediction of post-pubertal mandibular growth (17). Decision-making systems (DMS) and similar AI-based technologies demonstrate the advantages of knowledge-based approaches and DL, enabling automation and precise monitoring of orthodontic treatment progress. Furthermore, AI has shown the potential to objectively evaluate the effects of orthodontic treatment on facial appearance and estimate age-related changes, thereby providing a more comprehensive assessment of treatment outcomes (18).

The present study evaluated AI performance using a binary outcome (correct vs. incorrect landmark identification based on a predefined acceptance criterion) rather than continuous landmark coordinates. Consequently, agreement-based methods commonly used for continuous data, such as intraclass correlation coefficients (ICC), Bland–Altman analysis, and mean radial error, were not applicable to the statistical methods of this study. Instead, sensitivity, specificity, positive predictive value (PPV), and negative predictive value (NPV) were used because they are appropriate measures for evaluating the diagnostic performance of binary classification outcomes. This analysis was appropriate for assessing the AI model's ability to correctly classify landmark identification relative to the manual reference standard according to the predefined acceptance criterion. Future studies that evaluate continuous landmark coordinates or localization errors may incorporate agreement measures, including ICC, Bland–Altman analysis, mean radial error to provide a more comprehensive evaluation of AI-assisted cephalometric landmark identification.

The AI algorithm evaluated in the present study was designed to process data locally as well as within secure cloud-based environments that comply with standard security protocols. Such measures may help minimize data-related risks while supporting advancements in cephalometric analysis, diagnostic accuracy, and clinical efficiency. This study focused on the development and validation of an AI-based cephalometric analysis system using a DL model augmented by human expertise. The AI/human augmentation approach was intended to improve diagnostic accuracy through the integration of automated landmark identification with investigator-guided refinement. In addition, the inclusion of a broad range of cephalometric landmarks enabled a comprehensive evaluation of the AI model's ability to address the complexity and variability associated with cephalometric radiographs.

Identification of cephalometric landmarks and generation of measurements are often time-consuming and repetitive tasks for clinicians. Therefore, approaches that can accelerate this process while maintaining diagnostic accuracy would be beneficial. Recent advances in AI technologies have introduced new methods for automated cephalometric landmark detection and measurement generation. Evaluating the accuracy and reliability of these systems remains essential before widespread clinical implementation (11, 19). Since lateral cephalometric analysis represents a fundamental diagnostic tool in orthodontics, the present study aimed to develop an automated system for identifying cephalometric landmarks using a FPN trained to directly predict landmark coordinates. The findings demonstrated high concordance between manual tracing and the AI Ceph program, with concordance rates reaching approximately 96.6%, indicating the algorithm's strong ability to accurately identify the selected cephalometric landmarks.

Several previous studies have proposed algorithms for automated identification of landmarks on lateral cephalograms and have evaluated their accuracy (20–22). However, relatively few investigations have utilized DL algorithms specifically for automated landmark identification in lateral cephalograms (11, 23). The high accuracy achieved by the AI Ceph program in the present study may also be attributed to the careful selection of 18 hard tissue landmarks and one soft tissue landmark based on their clinical importance, reproducibility, ease of identification, uniformity of outline, and suitability for quantitative analysis.

A previous study conducted by Lindner in 2016 included 400 subjects aged 7–76 years. All cephalograms were manually traced by two experienced orthodontists, and a fully automated system was trained to identify 19 landmarks on lateral cephalograms. The reported average point-to-point error was 1.2 mm, with 84.7% of landmarks identified within the clinically accepted precision threshold of 2.0 mm (21). Similarly, in the present study, the maximum deviation between corresponding landmark locations identified by manual and AI tracing methods remained within 2 mm, supporting the consistency of the current findings with existing literature regarding AI accuracy. Nevertheless, the magnitude of discrepancy should be interpreted cautiously, as a 2 mm deviation may influence diagnosis and potentially alter treatment planning in certain clinical situations. Likewise, Hwang et al. reported a mean detection error of 1.46 ± 2.97 mm between AI and human examiners, which was comparable to the mean difference observed between human examiners themselves (1.50 ± 1.48 mm). Differences between AI and human examiners were generally less than 0.9 mm and were not considered clinically significant (24).

Early investigations comparing digital tracing systems with manual acetate-paper tracing methods demonstrated no significant differences in landmark identification accuracy (25–27). These studies also reported strong intra-examiner agreement between manual and computerized methods. However, inter-examiner repeatability remained limited with conventional manual tracing. Consistent with these findings, the current study demonstrated high concordance between manual tracing and the AI Ceph software for all selected landmarks. Only three landmarks, Sella (89.3%), Nasion (86.6%), and Glabella (89.5%), showed concordance values below 90%, whereas all remaining landmarks demonstrated concordance exceeding 90%.

More recent studies have focused on evaluating the accuracy of anatomical landmark recognition using various digital cephalometric software systems (23, 28, 29). Meric and Naoumova concluded that fully automated systems were capable of performing cephalometric analyses rapidly and accurately (29). Similarly, Bulatova et al. reported statistically significant differences for only a limited number of landmarks identified using automated digital cephalometric methods, although these differences were not clinically significant (23). In agreement with these findings, the present study identified statistically significant differences only for the Glabella landmark, whereas all other landmarks demonstrated no significant differences between AI and manual tracing methods. Importantly, the observed discrepancies were minimal and did not affect the overall diagnostic outcome, supporting the reliability and clinical applicability of the AI tracing method.

To further evaluate AI performance, a subset of cephalometric radiographs containing both correctly labelled and intentionally mislabelled landmarks was used during testing. Thirty-three landmarks were correctly labelled and an additional 33 landmarks were intentionally mislabelled. Sensitivity values exceeded 80% for most landmarks, while the Gonion landmark achieved 100% sensitivity, indicating that the AI system accurately identified Gonion in all evaluated radiographs. Comparison of concordance rates before and after training with mislabelled landmarks demonstrated no significant differences for most landmarks, except for Glabella, which showed a statistically significant improvement following training (*p* < 0.02). Several landmarks demonstrated slight increases in concordance following additional training. These findings are consistent with previous reports (29, 30) and suggest that AI performance may improve with continued training and exposure to additional datasets, reflecting the adaptive learning capability inherent to DL-based systems.

The future application of AI in orthodontic cephalometric analysis appears promising. Anticipated advancements include improved accuracy, integration of multimodal datasets, real-time clinical decision support, personalized treatment planning, and adaptive learning systems. Future AI algorithms are expected to incorporate advanced DL architectures and larger datasets to further improve the accuracy and specificity of cephalometric analysis and automated segmentation (31–33).

Despite the demonstrated accuracy and reliability of the tested AI algorithm, the present study has several limitations. First, the relatively small independent test set of cephalometric radiographs, which may limit the generalizability of the findings. Second, the study population included a broad age range which may have influenced landmark detection accuracy. Additionally, the evaluated system did not provide information regarding the directional variation of landmark discrepancies. Future studies using larger, multicenter external datasets should further validate the robustness and clinical applicability of the proposed AI model, evaluate its performance across specific age groups, and investigate landmark deviations in the sagittal and vertical dimensions, as these may influence the outcomes and interpretation of cephalometric analyses.

## 5 Conclusion

AI-assisted detection of cephalometric landmarks demonstrated high agreement and concordance with conventional manual tracing methods. Although minor variations were observed in the location of certain landmarks, these discrepancies did not exceed 2 mm and were considered clinically acceptable. Nevertheless, AI-based cephalometric tracing systems should be implemented cautiously before considering replacement of conventional manual tracing methods. Currently available AI applications for cephalometric landmark identification still require clinician oversight to verify the accuracy of landmark positioning.

## Data Availability

The raw data supporting the conclusions of this article will be made available by the authors, without undue reservation.
